# Effect of Metal Elements on Microstructure and Mechanical Properties of Ultrafine Cemented Carbide Prepared by SPS

**DOI:** 10.3390/molecules29071678

**Published:** 2024-04-08

**Authors:** Hao Jiang, Siyuan Fu, Zichang Zhang, Shun Wang, Zhiwei Zhao

**Affiliations:** College of Materials Science and Engineering, Henan University of Technology, Zhengzhou 450001, China; jhao202210@163.com (H.J.); siyuan010702@163.com (S.F.); z2975817695@163.com (Z.Z.); shun_wang@haut.edu.cn (S.W.)

**Keywords:** cemented carbide, spark plasma sintering, metal elements, microstructure, mechanical properties

## Abstract

To satisfy the needs of precision machining, ultrafine tungsten carbide (WC)-based cemented carbide with fine grain size and excellent mechanical properties was prepared. Ultrafine cemented carbide was prepared by spark plasma sintering (SPS) using WC, Co as raw materials and metal elements V, and Cr as additives, and the effects of metal elements on the microstructure and mechanical properties of cemented carbide were investigated. The results show that the specimen (91.6WC-1.2V-1.2Cr-6Co) prepared at 1350 °C, 6 min, 25 MPa has the best mechanical properties (*H_V_* 2322.9, *K_IC_* 8.7 MPa·m^1/2^) and homogeneous microstructure. The metal elements could react with WC to form a (W, V, Cr) Cx segregation layer, which effectively inhibits the growth of WC grains (300 nm). The combination of SPS and metal element additives provides a new approach for the preparation of ultrafine cemented carbides with excellent properties.

## 1. Introduction

Cemented carbides are composed by the hard phase refractory metal carbide WC and the bonding phase Co [1,2]. Owing to its excellent hardness, fracture toughness, wear resistance, and high temperature stability, it was widely used in machining, drilling, and mining applications [3,4,5,6]. In the last century, the hardness and wear resistance of WC–Co cemented carbides have been put to a higher test in order to meet the growing demands in the field of high-speed and precision machining. Research has shown that the properties of cemented carbide make a quantum leap as the alloy grain size is reduced to the micrometer or nanometer scale [7,8,9]. Therefore, the preparation of ultrafine or nanoceramics with excellent properties has attracted worldwide attention [10,11,12].

Adopting rapid sintering technology and adding grain growth inhibitors (GGIs) are effective methods to prepare ultrafine cemented carbides. Rapid sintering methods included spark plasma sintering (SPS) [13,14], microwave sintering [15,16], selective laser sintering [17,18], high-frequency induction sintering [19,20], and electric heating methods [21,22,23]. Spark plasma sintering has a special sintering mechanism [24,25,26,27], which can effectively reduce the sintering temperature, shorten the sintering time, and improve the degree of densification of the specimen. In addition, GGIs are very effective in inhibiting the WC grain growth process. Transition metal carbides such as VC [28], Cr_3_C_2_ [29], TiC [30], TaC [31], and NbC [32] are the most commonly used grain growth inhibitors and are added into the matrix by ball milling and mixing. However, the effect of metal elements on the microstructure and mechanical properties of cemented carbides has rarely been investigated.

In this study, ultrafine cemented carbides containing metal elements were prepared by SPS using nano-WC and nano-Co powders as raw material and metal elements V and Cr as additives. Compared with the traditional sintering method, spark plasma sintering has a special sintering mechanism [24,25,26,27] which can accelerate the diffusion rate and realize the densification of samples at a lower sintering temperature and shorter holding time. Nanopowders have a large specific surface area and high reaction activity, which can react at lower sintering conditions [33]. The combination of the two can effectively inhibit the growth of WC grains. In this work, ultrafine cemented carbide was prepared by spark plasma sintering combined with the addition of metal elements, the effects of the content of metal elements added, and the sintering temperature, holding time, and sintering pressure on the microstructure and mechanical properties of cemented carbide were investigated.

## 2. Results

### 2.1. TG-DSC Analysis Results

**Figure 1:**
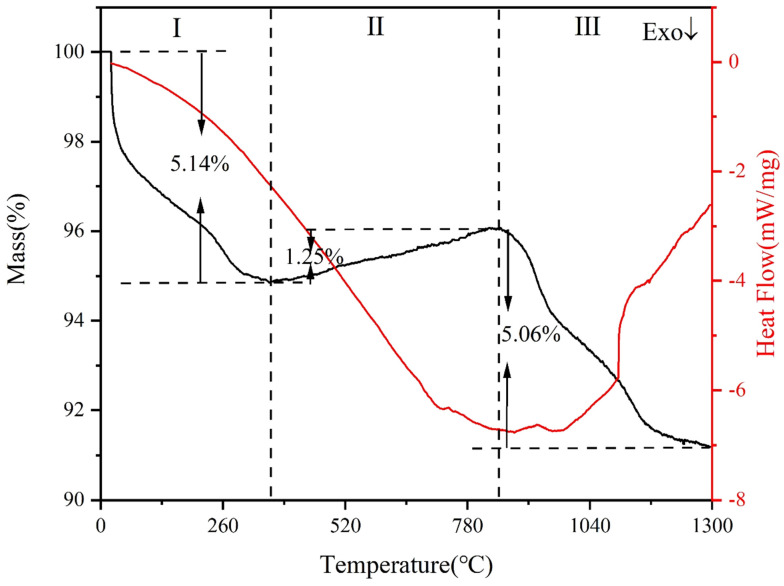
TG-DSC curves of the specimen at an addition rate of x = 1.2 wt.%.

To determine the possible reactions during spark plasma sintering, TG-DSC measurements were carried out on the mixture with x = 1.2 wt.%, and the results are shown in Figure 1. As shown in Figure 1, the mass loss of the raw material was 5.14% in stage I (room temperature—362 °C). This is mainly due to the escape of moisture adsorbed on the surface of the powder [34]. In stage II (362–846 °C), the mass of the powder increased by 1.25%. This may be attributed to the reaction between the powder and the oxygen adsorbed on its surface, resulting in a slight increase in the powder mass. Although the heating was carried out under the protection of the argon atmosphere throughout, some of the finer sized powders were still oxidized due to the higher reactivity. In stage Ⅲ (846 °C–1300 °C), the powder mass loss was 5.06%. This may be due to the carbothermal reduction reaction between the oxidized powder and the free carbon in the matrix at this heating stage, resulting in a decrease in the quality of the raw material.

From the DSC curve (Figure 1), it showed that an exothermic reaction occurs in the range of room temperature to 740 °C. The following reactions may be occurring: 4V(s) + 5O_2_(g) = 2V_2_O_5_(s); 4Cr(s) + 3O_2_(g) = 2Cr_2_O_3_(s). A small heat absorption peak at 740 °C may be due to the following transformation [35]: Cr_2_O_3_(s) + C(s) = 2CrO(s) + Co(g)↑. An absorption peak appears in the range of 880 °C–970 °C, which may be due to the following transformations [36,37,38]: V_2_O_3_→V_8_C_7_, Cr_2_O_3_→Cr_3_C_2_. The carbonation of V and Cr elements proceeds in large quantities under the conditions of 970 °C–1300 °C. The melting onset (Tm) and melting peak (Tp) values of each reaction that may occur during the heating process of the raw material are shown in Table 1.

### 2.2. Phase Composition and Bonding State

**Figure 2:**
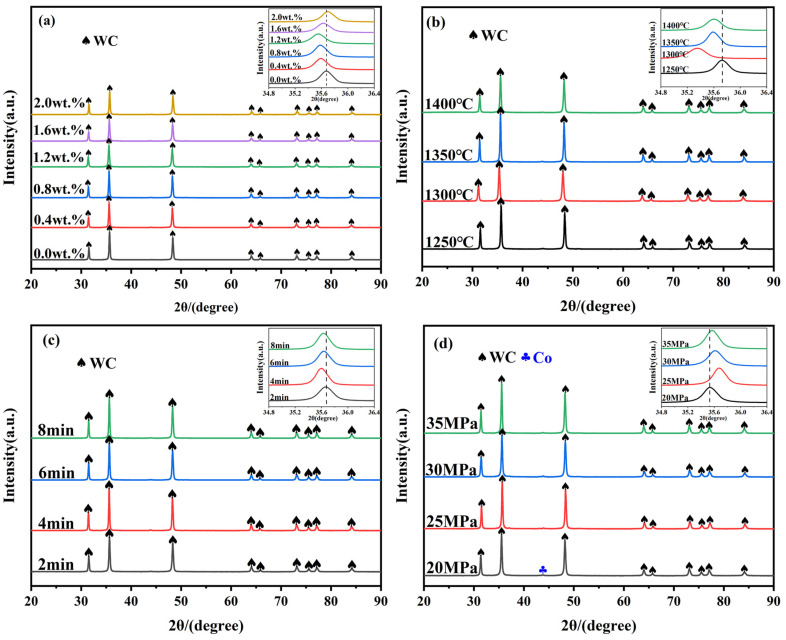
XRD patterns of cemented carbides containing different contents of metal elements prepared by SPS under different sintering conditions: (**a**) metal element content (1300 °C, 4 min, 30 MPa), (**b**) sintering temperature (1.2 wt.%, 4 min, 30 MPa), (**c**) holding time (1.2 wt.%, 1350 °C, 30 MPa), (**d**) sintering pressure (1.2 wt.%, 1350 °C, 6 min).

Figure 2 shows the XRD patterns of cemented carbide containing different contents of metal elements prepared by SPS under different sintering conditions.

Figure 2a shows the XRD pattern of the specimens at the same sintering temperature (1300 °C), the same holding time (4 min), the same sintering pressure (30 MPa), and different metal element contents (x = 0.0–2.0 wt.%). As shown in Figure 2a, the XRD pattern is composed mainly of WC diffraction peaks and no diffraction peaks of V and Cr were found. This is mainly attributed to the relatively small additions of V and Cr. The intensity of the WC diffraction peaks shows a tendency of increasing and then decreasing with the increase in the addition of metal elements. When the addition amount is x = 0.8–1.2 wt.%, the intensity of WC diffraction peaks reaches the maximum. This is mainly due to the fact that V and Cr gradually enter into the lattice interstitials of WC with the increase in the addition amount, forming the (W, V, Cr) Cx segregation layer at the interface of WC grains. The existence of the segregation layer inhibited the dissolution–precipitation process of the WC grains, resulting in an increase in the intensity of the WC diffraction peaks. With the further increase in the addition amount, the solubility of V and Cr in the liquid-phase Co reached the maximum, and the dissolution–precipitation process could not be further inhibited, so part of the WC grains were dissolved into the liquid-phase Co, and the intensity of the WC diffraction peaks decreased. In addition, the WC diffraction peaks are first shifted to a small angle and then to a large angle with the increase in metal addition. From the Bragg’s equation (2*d* sin*θ* = k*λ*), the crystal plane spacing first increases and then decreases. This is mainly attributed to the fact that with the increase in addition, V and Cr atoms enter the lattice interstitials of WC, resulting in an increase in the crystal plane spacing. With a further increase in the addition amount, V and Cr precipitated out after reaching the maximum solubility and the crystal plane spacing decreased [39].

Figure 2b demonstrates the XRD patterns of the specimens with the same metal element addition (x = 1.2 wt.%), the same holding time (4 min), the same sintering pressure (30 MPa), and different sintering temperatures (1250–1400 °C). As shown in Figure 2b, the intensity of WC diffraction peaks showed a trend of decreasing, then increasing, and then decreasing, with an increase in the sintering temperature. The intensity of the WC diffraction peak at 1300 °C is slightly lower than that at 1250 °C, which may be attributed to the fact that a large amount of liquid-phase Co is produced at this temperature, which promotes the dissolution–precipitation process of WC, and the intensity of the diffraction peaks thus decreases. When the sintering temperature is 1350 °C, the intensity of WC diffraction peaks reaches the maximum. This may be due to the diffusion and reaction of V and Cr atoms occurring more sufficiently at this temperature, forming a large amount of the (W, V, Cr) Cx segregation layer which inhibits the dissolution of WC grains [40]. When the sintering temperature was 1400 °C, the intensity of the WC diffraction peaks slightly decreased. In addition, the WC diffraction peaks show a tendency to shift to a small angle. This is mainly owing to the increase in the sintering temperature, which helps the diffusion and reaction of metal elements to form more (W, V, Cr) Cx segregation layers.

In this experiment, the temperature at the beginning of liquid-phase sintering is much lower than that of the traditional sintering method [41,42] (>1400 °C). This is mainly attributed to the special sintering mechanism of spark plasma sintering [28]: the concentration of the current formed at the contact point between particles leads to the temperature at this location being much higher than the sample volume temperature, which can cause the powder Co to melt and produce the liquid phase. In addition, the particle size at the nanoscale level increases the surface reactivity and helps to prepare specimens at lower sintering temperatures. The same experimental phenomenon was also observed in the study of N. Rezlescu [43].

Figure 2c exhibits the XRD patterns of the specimens at the same metal element addition (x = 1.2 wt.%), the same sintering temperature (1350 °C), the same sintering pressure (30 MPa), and different holding times (2–8 min). As shown in Figure 2c, the WC diffraction peaks showed a tendency to increase first (2–4 min) and then remain stable (4–8 min) with the increase in holding time. This is due to the diffusion of V and Cr atoms and the reaction with WC being more sufficient with the prolongation of the holding time, and the formation of the (W, V, Cr) Cx segregation layer effectively inhibits the dissolution–precipitation process of WC. Meanwhile, the WC diffraction peaks show an overall tendency to shift to a small angle, which is mainly due to the formation of the segregation layer to change the crystal plane spacing.

Figure 2d shows the XRD patterns of the specimens at the same metal element addition (x = 1.2 wt.%), the same sintering temperature (1350 °C), the same holding time (6 min), and different sintering pressures (20–35 MPa). As shown in Figure 2d, the phase composition of the specimen is mainly WC and Co. As the sintering pressure increases, the peak for the Co phase disappears. This is mainly because the flow of Co is not sufficient at lower sintering pressures and is prone to agglomeration. With the increase in sintering pressure, the viscous flow of liquid-phase Co is more adequate and the agglomeration phenomenon disappears. The intensity of the WC diffraction peaks is relatively strong at 25 MPa and 35 MPa. This is mainly because at 25 MPa, the viscous flow of liquid-phase Co is more adequate, the V and Cr atoms diffuse more uniformly with the liquid-phase Co, and the formation of the segregation layer effectively inhibits the dissolution–precipitation process of WC grains. When the sintering pressure was 35 MPa, the excessive pressure resulted in the liquid-phase Co being extruded from between the WC grains. The diffusion distance of W and C elements was shortened, the chances of direct contact between the WC grains were elevated, and the WC grains abnormally grew [44].

In conclusion, the WC diffraction peak reached its maximum value at an addition amount of 1.2 wt.%, which indicated that a sufficient number of polarization layers could be formed in the matrix at an addition amount of X = 1.2 wt.%. When the sintering temperature reaches 1350 °C, the intensity of the WC diffraction peak reaches a larger value, which indicates that the amount of liquid-phase Co in the matrix is further increased, the flow is more adequate, and the V and Cr dissolved therein are able to diffuse and react more adequately and inhibit the dissolution–precipitation of WC grains. The prolongation of the holding time and the increase in the sintering pressure can play a role in promoting the liquid-phase Co and the dissolved V and Cr to realize the fuller diffusion and reaction.

**Figure 3:**
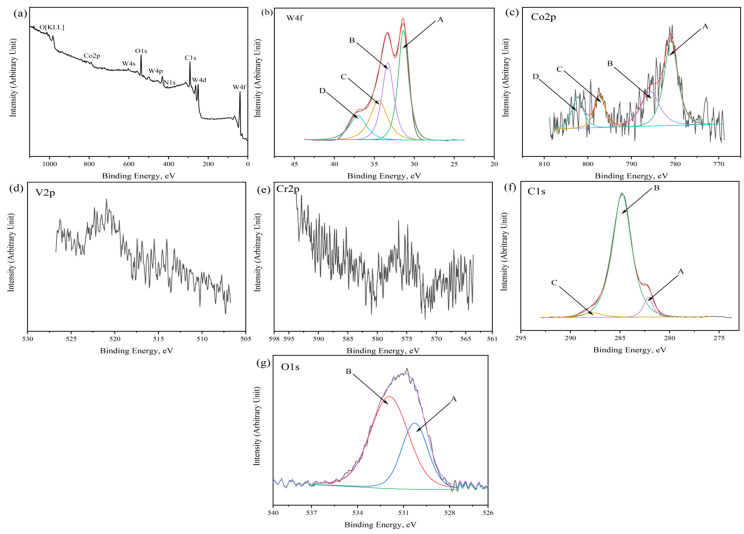
XPS spectra of WC-based cemented carbide (WC-1.2V-1.2Cr-Co): (**a**) full spectrum; (**b**) W4f; (**c**) Co2p; (**d**) V2p; (**e**) Cr2p; (**f**) C1s; (**g**) O1s.

In order to further investigate the chemical composition and bonding state of the specimens, the specimens (WC-1.2V-1.2Cr-Co) prepared under the conditions of 1350 °C, 6 min, and 25 MPa were measured by XPS, and the results of the tests are shown in Figure 3:

As shown in Figure 3, the sample surface mainly consists of W, Co, V, Cr, and O elements. As shown in Figure 3b, peak A (31.32 eV) is attributed to WC of W4f_7/2_ species and peak B (33.37 eV) corresponds to WO_3_ of W4f_7/2_. Peak C (34.82 eV) and peak D (37.21 eV) correspond to the other carbides of W. Figure 3c shows the XPS spectrum of Co2p. Peak A (781.05 eV) corresponds to Co_2_O_3_. This is attributed to the fineness of Co powder, which is easily oxidized during preparation and sintering [45,46]. Peak B (785.39 eV), peak C (796.78 eV), and peak D (803.36 eV) belong to other compounds of Co. The peak with a binding energy of 520.95 eV corresponds to a carbocation of V of the V2p_3/2_ species, as shown in Figure 3d. The peak with a binding energy of 585.3 eV is attributed to the carbides of Cr, as shown in Figure 3e. As shown in Figure 3f, peak A (282.06 eV) belongs to WC and peak B (284.82 eV) is assigned to free carbon. Peak C (287.8 eV) corresponds to C=O. The reason may be that residual pump oil small molecules in the cavity of the XPS detection equipment deposit on the surface of the sample during the vacuum pumping process, leading to the appearance of peak C. As shown in Figure 3g, peak A (530.30 eV) belongs to Cr_2_O_3_ and peak B (531.90 eV) attributes to OH, which is mainly due to the small amount of water absorbed on the surface of the specimen [47,48].

As shown by the results of XPS analysis of each peak, the carbides of V and Cr were successfully synthesized in the matrix. These carbides play a critical role in the WC grain refinement process. This also corroborates with the XRD results that the carbides formed by V and Cr with free C atoms in the matrix affected the position and intensity of the WC diffraction peaks.

### 2.3. Microstructure

**Figure 4:**
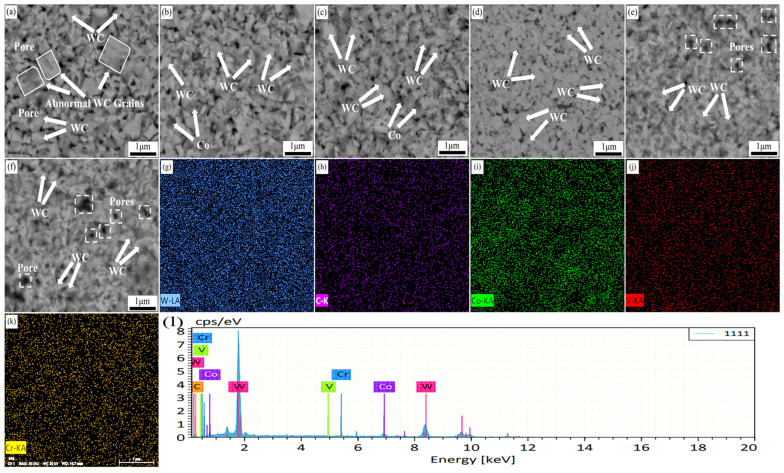
Backscattering images, mapping images, and EDS spectra of specimens with different metal element contents under the same sintering conditions (1300 °C, 4 min, 30 MPa): (**a**) x = 0.0 wt.%; (**b**) x = 0.4 wt.%; (**c**) x = 0.8 wt.%; (**d**) x = 1.2 wt.%; (**e**) x = 1.6 wt.%; (**f**) x = 2.0 wt.%; (**g**) element W; (**h**) element C; (**i**) element Co; (**j**) element V; (**k**) element Cr; (**l**) EDS spectrum.

Figure 4 demonstrates the SEM backscattering images, mapping, and EDS energy spectra of the specimens with different metal element additions at the same sintering parameters (1300 °C, 4 min, and 30 MPa).

As shown in Figure 4a, the average grain size of WC was larger (400–600 nm) when the addition amount was x = 0.0 wt.%, and some WC grains were found to grow abnormally (1 μm) in the matrix. When the addition amount is x = 0.4–0.8 wt.%, the WC grain size is significantly refined and the abnormal growth phenomenon is suppressed. This is mainly attributed to the increasing number of (W, V, Cr) Cx segregation layers formed with the increasing amount of V and Cr elements added. The formation of the segregation layer inhibits the dissolution–precipitation process and realizes the refinement of the WC grains. The WC grain size was minimized (300–400 nm) when the addition amount was x = 1.2 wt.%. At this time, the specimen has a uniform microstructure and fine WC grain size. This indicates that the appropriate metal element addition helps to refine the WC grains, optimize the microstructure, and enhance the densification of the specimen. With further increase in the addition amount (x = 1.6–2.0 wt.%), the WC grain size further decreases (200–300 nm). However, a large number of pores and defects appear in the matrix. This is mainly attributed to the existence of the segregation layer; although it can inhibit the growth of WC grain size, it also reduces the interfacial coherence between WC and Co, which leads to the appearance of pores and defects [49,50,51]. The mapping (Figure 4g–k) of the specimen with an addition of x = 1.2 wt.% shows that the elements are uniformly distributed in the specimen. The EDS spectra (Figure 4l) indicate that the selected region is mainly composed of the elements W, C, and Co, with small amounts of the elements V and Cr.

**Figure 5:**
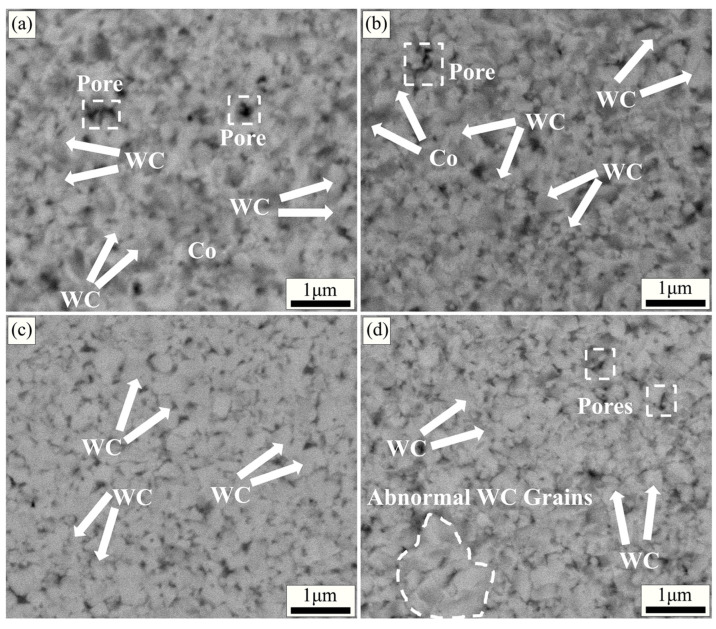
Backscattering images of specimens at different sintering temperatures (x = 1.2 wt.%, 4 min, 30 MPa): (**a**) 1250 °C; (**b**) 1300 °C; (**c**) 1350 °C; (**d**) 1400 °C.

**Figure 6:**
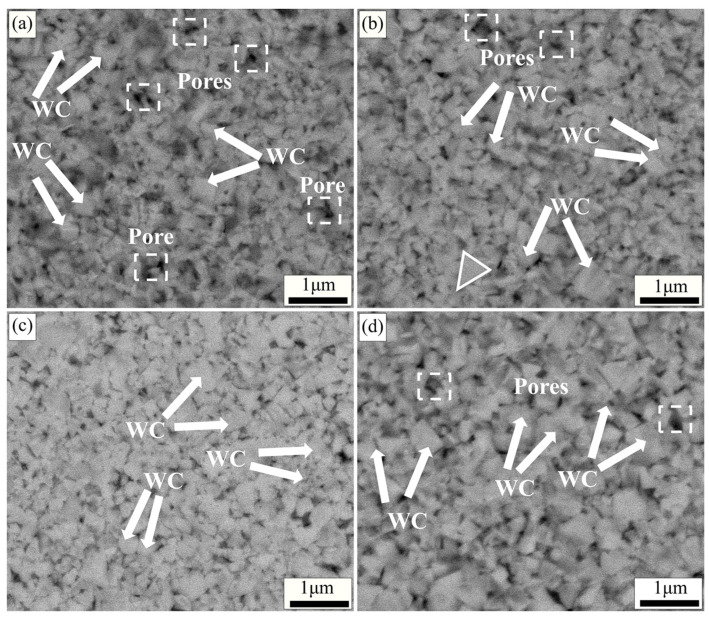
Backscattering images of specimens at different holding times (x = 1.2 wt.%, 1350 °C, 30 MPa): (**a**) 2 min; (**b**) 4 min; (**c**) 6 min; (**d**) 8 min.

**Figure 7:**
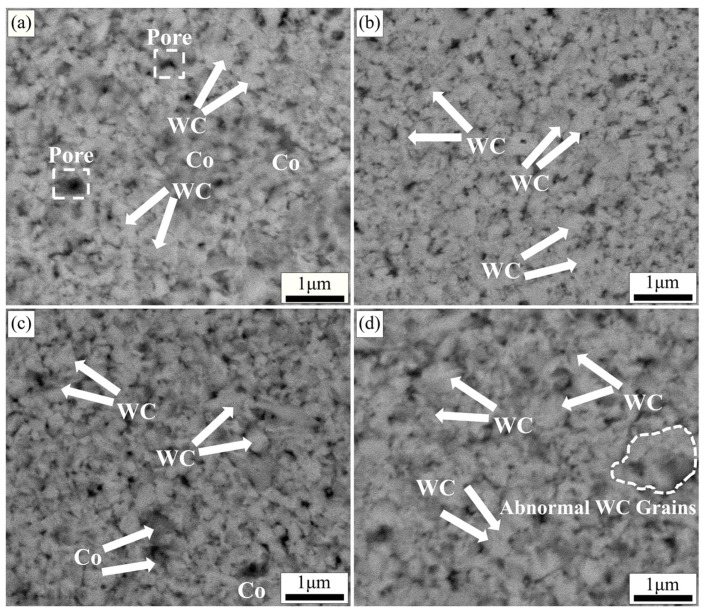
Backscattering images of specimens at different sintering pressures (x = 1.2 wt.%, 1350 °C, 6 min): (**a**) 20 MPa; (**b**) 25 MPa; (**c**) 30 MPa; (**d**) 35 MPa.

Figure 5, Figure 6 and Figure 7 use the same EDS and Mapping techniques as in Figure 4, and the results similarly demonstrate a uniform distribution of elements in the matrix.

Figure 5 demonstrates the backscattering images of specimens with different sintering temperatures at the same metal element addition, same holding time, and same sintering pressure (x = 1.2 wt.%, 4 min, 30 MPa). As shown in Figure 5a, when the temperature is 1250 °C, the grain size distribution of WC is not uniform, and some pores and defects exist in the matrix. This is mainly due to the inhomogeneous matrix microstructure caused by an insufficient viscous flow of liquid-phase Co at a lower sintering temperature. When the sintering temperature was 1300 °C (Figure 5b), it can be seen that the WC grains were significantly refined (around 400 nm). This is attributed to the more adequate viscous flow of Co with increasing temperature, which promotes the diffusion of V and Cr atoms. The formation of more (W, V, Cr) Cx segregation layers facilitates the refinement of WC grains. As shown in Figure 5c, when the sintering temperature was 1350 °C, the microstructure of the specimen was more uniform and the WC grain size was smaller (around 300 nm). There is no obvious pores and defects in the matrix at this temperature. That indicates that the suitable sintering temperature helps the viscous flow of Co and the rearrangement of particles, which enhances the densification of the specimen [52]. At this sintering temperature, the diffusion and reaction of both V and Cr atoms are more adequate, and the growth of WC grains in the matrix is uniformly suppressed. At this sintering temperature, the degree of densification and WC grain size of the specimen reached an equilibrium. When the sintering temperature was raised to 1400 °C (Figure 5d), an abnormal growth of WC grains appeared in the matrix. This is mainly owing to the over-high sintering temperature that will cause the evaporation of liquid-phase Co, which leads to the shortening of the diffusion distance of W and C atoms.

Figure 6 demonstrates the backscattering images of the specimens with different holding times at the same metal element addition, same sintering temperature, and same sintering pressure (x = 1.2 wt.%, 1350 °C, 30 MPa). As shown in Figure 6a, a large number of pores and defects exists in the specimen at a holding time of 2 min. This is mainly attributed to the small amount of liquid-phase Co at the shorter holding time, which cannot adequately fill and heal the pores and defects in the matrix. When the holding time was 4 min (Figure 6b), the WC grains in the matrix appeared to be obviously refined, and some grains are triangular prismatic. As known from the literature [40,53], the appearance of trigonal grains indicates the appearance of the (W, V, Cr) Cx segregation layer, and the growth of WC grains is inhibited. At this time, the size of WC grains in the matrix is not uniform, and some pores and defects still exist, which indicates that the holding time is not sufficient. As shown in Figure 6c, the WC grain size distribution is more uniform and fine (350 nm) when the holding time is 6 min. There is no obvious large number of pores and defects in the matrix. This indicates that a suitable holding time helps the refinement of WC grains and the homogeneity of microstructure. When the holding time is 8 min (Figure 6d), the WC grains grow abnormally, and some WC grain sizes reach 500 nm.

Figure 7 presents the backscattering images of specimens with different sintering pressures at the same metal element addition, same sintering temperature, and same holding time (x = 1.2 wt.%, 1350 °C, 6 min). As shown in Figure 7a, the WC grain size is finer when the sintering pressure is 20 MPa. However, there are more pores and defects in the matrix. When the sintering pressure is 25 MPa (Figure 7b), the densification of the specimen is high and the microstructure is uniform. The WC grain size is around 300 nm. This is mainly attributed to how the liquid-phase Co can fully fill the pores between the WC grains by capillary force and viscous flow under suitable sintering pressure [12]. Meanwhile, the V and Cr atoms dissolved in the liquid-phase Co can be more uniformly distributed in the matrix, and the (W, V, Cr) Cx segregation layer generated by the reaction with WC can have an overall uniform inhibition effect on the WC grains in the matrix. With the further increase in sintering pressure (30–35 MPa), as shown in Figure 7c,d, the WC grain size increases significantly. This is mainly due to the high sintering pressure, which causes the liquid-phase Co in the particle gap to be squeezed out [44,54], and the probability of the direct contact of WC particles increases, resulting in an increase in grain size.

### 2.4. Mechanical Properties

**Figure 8:**
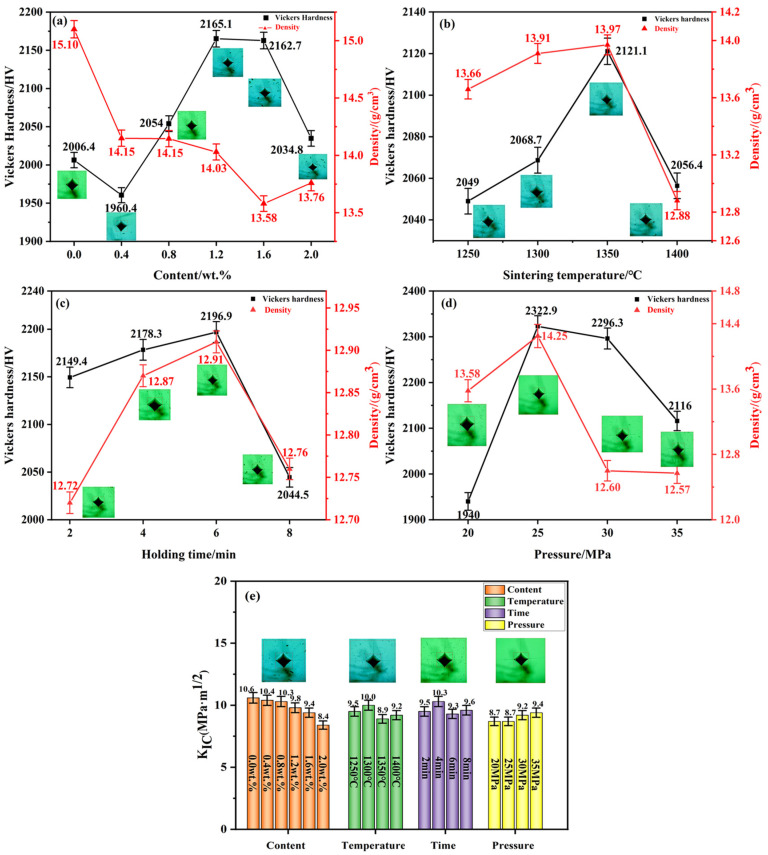
Mechanical properties of cemented carbides prepared using SPS under different conditions: (**a**) metal element content; (**b**) sintering temperature; (**c**) holding time; (**d**) sintering pressure; (**e**) fracture toughness of specimens.

Figure 8 shows the mechanical properties of different specimens prepared using SPS under different conditions.

As shown in Figure 8a, the Vickers hardness of the specimen shows a trend of decreasing, then increasing, and then decreasing, with the increase in the addition of metal elements. The Vickers hardness of cemented carbide decreases when the addition amount is x = 0.4 wt.%. This is mainly due to the good interfacial bonding between WC and Co in the cemented carbide without the addition of metal elements [55]. The microstructure is more homogeneous and the amount of pores and defects is less, as shown in Figure 4a. With the increase in addition, the number of (W, V, Cr) Cx segregation layers is increasing, the WC grain size decreases gradually (Figure 4b–e), and the hardness increases. The Vickers hardness, density, and fracture toughness of the specimens were *Hv* 2165.1, 14.03 g/cm^3^, and 9.8 MPa·m^1/2^, respectively, when the additive amount was x = 1.2 wt.%. In this case, the specimens had fine grain sizes (200–300 nm) and a homogeneous microstructure. The density value of the specimen showed a trend of gradual decrease. This is mainly attributed to the formation of a segregation layer in the WC matrix with increasing metal element additions, which leads to the weakening of the WC/Co interfacial bonding, with the consequent appearance of pores and defects [49]. The density and the homogeneity of the microstructure of the specimen are thus affected.

As shown in Figure 8b, the Vickers hardness and density of the specimens showed an increasing and then decreasing trend as the sintering temperature increased. With the increase in sintering temperature, the content of liquid-phase Co in the specimen is increasing, and the densities of the specimens are improving. The hardness density and fracture toughness of the specimens were *Hv* 2121.1, 13.97 g/cm^3^, and 8.90 MPa·m^1/2^ at the sintering temperature of 1350 °C. Compared with that at 1250 °C (*Hv* 2049, 13.66 g/cm^3^ and 9.50 MPa·m^1/2^), the hardness and density increased by 3.52% and 2.27%, respectively. However, the fracture toughness has decreased. This is mainly indicated that at the lower sintering temperature (1250 °C), a sufficient number of (W, V, Cr) Cx-polarized layers have not been formed in the alloy matrix and the WC grain size is still relatively large. The fracture mode of this alloy is transgranular fracture [12,51]. Transgranular fracture can effectively inhibit the crack propagation and improve the fracture toughness of the sample. As the sintering temperature increases, the V and Cr diffusion rate accelerates and an increasing number of segregation layers begin to form. While the size of WC grains is reduced, the propagation form of cracks in the matrix is also changed from transgranular fracture to intergranular fracture. Cracks are generated and propagated along the grain boundaries, and the fracture toughness of the sample decreases [56,57].

As shown in Figure 8c–e, the hardness and fracture toughness of the specimens showed the same trend of increasing and then decreasing with the increase in holding time and sintering pressure. The hardness, density, and fracture toughness of the specimens at 1350 °C, 6 min, and 25 MPa were *Hv* 2322.9, 14.25 g/cm^3^, and 8.70 MPa·m^1/2^, respectively. Compared with that of 20 MPa (*Hv* 1940, 13.58 g/cm^3^, and 8.7 MPa·m^1/2^), the hardness and density of the specimens were enhanced by 19.74% and 4.93%, respectively, and the fracture toughness was unchanged. This indicates that the appropriate holding time and sintering pressure help to enhance the mechanical properties and densification of the specimens. An excessive holding time and sintering pressure will lead to the growth of WC grains and inhomogeneity of microstructure, and the mechanical properties of the specimens are thus affected [54,58]. The mechanical property data of the specimens corresponded well with the backscattering diagrams (Figure 4, Figure 5, Figure 6 and Figure 7).

### 2.5. Magnetic Properties

**Figure 9:**
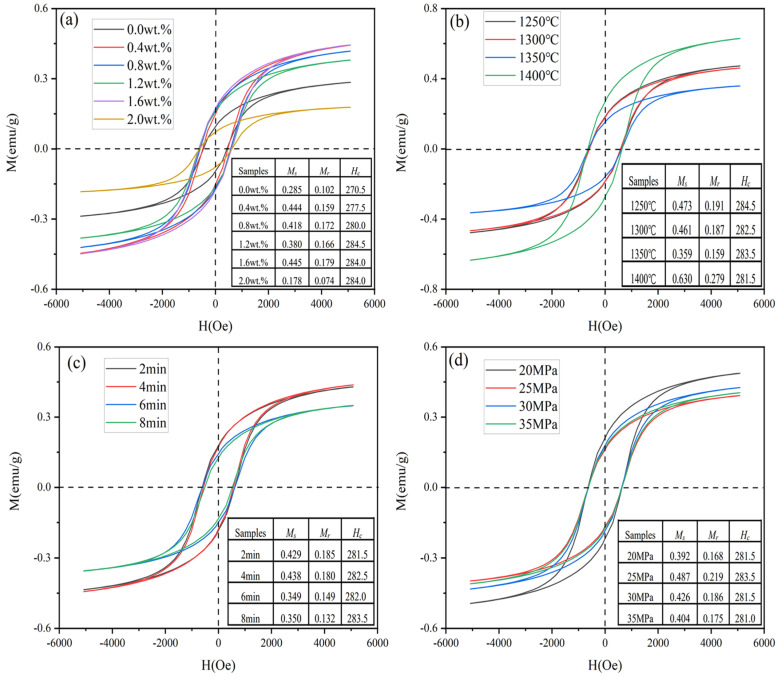
Hysteresis loops and magnetic properties of WC-based cemented carbides prepared by SPS under different conditions: (**a**) content; (**b**) sintering temperature; (**c**) holding time; (**d**) sintering pressure.

Figure 9 demonstrates the hysteresis loops and magnetic properties of WC-based cemented carbides prepared by SPS under different conditions. As shown in Figure 9a, when the metal element content is x = 0.4–1.6 wt.%, the sample has a relatively high saturation magnetization *M_s_* (0.380–0.445 emu/g) and remanent magnetization *M_r_* (0.159–0.179 emu/g). According to the ferromagnetic energy band theory [59,60], some of the valence electrons of solute atoms such as W dissolved in Co are transferred to the empty 3*d* orbitals of cobalt atoms, which has an effect on the magnetic interactions between cobalt atoms. With the increase in the addition amount, V and Cr atoms enter into the lattice of WC and form the (W, V, Cr) Cx segregation layer at the interface. The presence of the segregation layer inhibits the diffusion of W atoms into the liquid-phase Co, and the magnetic properties of the specimen are thus improved [61]. Studies have shown that the coercivity *H_c_* is closely related to the size of WC grains [62]. When the metal addition amount is x = 1.2 wt.%, the sample has the smallest grain size (Figure 4) and the best mechanical properties (Figure 8), and at this time, the sample also has the largest coercivity *H_c_* 284.5 Oe. This indicates that the grain size of WC is effectively suppressed at the appropriate addition amount, and the WC grain size is small, and the mechanical properties of the sample are excellent. The microstructure, mechanical properties, and magnetic properties corroborate each other.

Figure 9b shows the hysteresis loops and magnetic properties of the specimens at different sintering temperatures. With the increase in sintering temperature, the magnetic properties of the specimens showed an overall trend of decreasing and then increasing. Notably, at the sintering temperature of 1350 °C, the grain size of the specimen is smaller (Figure 5), the mechanical properties are also higher (Figure 8), and the *H_c_* also reaches a large value of 283.5 Oe. As shown in Figure 9c, the coercivity *H_c_* of the specimen reaches 282.0 Oe at a holding time of 6 min. The magnetic properties of the specimen are maximized at a sintering pressure of 25 MPa with *M_s_*, *M_r_*, and *H_c_* of 0.487 emu/g, 0.219 emu/g, and 283.5 Oe, respectively. This indicates that the proper sintering pressure contributes to the diffusion and reaction of V and Cr. At this time, the WC grain size is fine and the specimen has better magnetic properties. The magnetic properties correspond well to the XRD (Figure 2d) and backscatter diagrams (Figure 7b).

### 2.6. Comparison of Mechanical Properties

**Figure 10:**
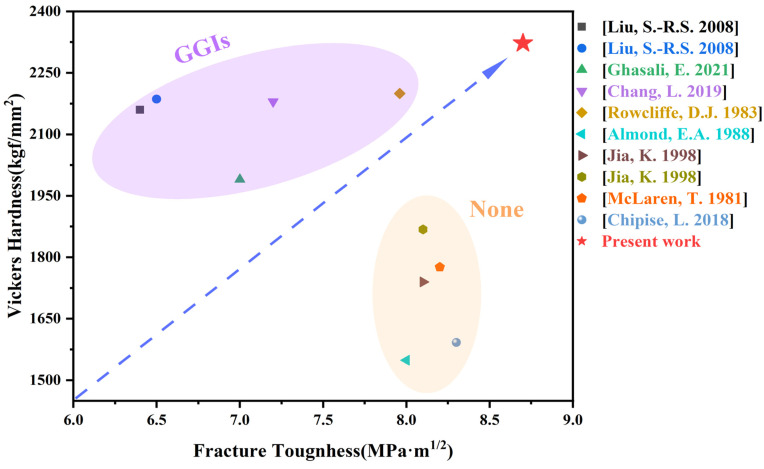
Mechanical properties comparison of different types of cemented carbide, [3]: Chang, L. 2019; [63]: Liu, S.-R.S. 2008; [64]: Ghasali, E. 2021; [65]: Rowcliffe, D.J. 1983; [66]: Almond, E.A. 1988; [67]: Jia,K. 1998; [68]: McLaren, T. 1981; [69]: Chipise, L. 2018.

Figure 10 demonstrates the comparison of the mechanical properties of different types of cemented carbides [3,63,64,65,66,67,68,69].

As shown in Figure 10, the hardness of the tungsten carbide with the addition of GGIs is in the range of 1990–2200 and the fracture toughness is in the range of 6.4–8.0. The hardness of cemented carbide without added GGIs is in the range of 1549–1868 and fracture toughness is in the range of 8.0–8.3. In general, cemented carbides that combine high hardness and high fracture toughness are rare. Compared with the other two cemented carbides, the cemented carbide prepared in this study with added metal elements has more excellent mechanical properties (*Hv* 2322.9, *K_IC_* 8.7 MPa·m^1/2^). This is mainly due to the faster diffusion of metal elements under SPS, which can be more uniformly distributed in the matrix, refine the WC grains, optimize the microstructure, and enhance the mechanical properties of the specimens.

## 3. Materials and Methods

### 3.1. Specimen Preparation

In this experiment, nano WC (99.9%, 200 nm, Shanghai Shuitian, Shanghai, China), nano Co (99.9%, 50 nm, Shanghai Shuitian, Shanghai, China), nano V (99.9%, 1–3 μm, Shanghai Shuitian, Shanghai, China), and nano-Cr (99.9%, 1–3 μm, Shanghai Shuitian, Shanghai, China) were used as raw materials for sample preparation according to the ratio of (94 − 2x)WC-xV-xCr-6Co (x = 0.0 wt.%, 0.4 wt.%, 0.8 wt.%, 1.2 wt.%, 1.6 wt.%, 2.0 wt.%). The composition and elemental content of the components in the experiment are shown in Table 2. The melting point of each raw material is shown in Table 3. The raw materials were ball milled in a QM-3SP2 planetary ball mill (Nanjing Laibu Technology Industry Co., Ltd., Nanjing, China) with a ball to material ratio of 5:1, a rotational speed of 180 rmp, and a ball milling medium of anhydrous ethanol. The ball milling jar and balls were made of cemented carbides. After 24 h of ball milling, the mixture was dried under vacuum at 80 °C for 12 h. The dried powders were sintered in the SPS-30 SPS sintering furnace (Shanghai Chenxin Electric Furnace Co., Ltd., Shanghai, China). From previous studies [70], the preparation of ultrafine cemented carbide by spark plasma sintering can significantly accelerate the sintering process and promote the evolution of the microstructure. Therefore, in order to study the evolution of the microstructure of the specimen during the sintering process, the sintering temperature range of 1250–1400 °C was determined. The use of a smaller sintering pressure and shorter holding time can significantly inhibit the abnormal growth of WC grains. Thus, the holding times were 2, 4, 6, and 8 min, and the sintering pressures were 20, 25, 30, and 35 MPa. During the sintering process, the vacuum pressure was 10^−2^ Pa and the heating rate was 100 °C/min. The sintered specimen is cut into *Φ* 10 mm × 5 mm strips by wire cutting.

### 3.2. Characterization

Thermogravimetry and differential scanning calorimetry (TG-DSC) of the powders were carried out using a DSC2003 thermogravimetric analyzer (NETZSCH, Bavaria, Germany) in a constant argon atmosphere with a heating rate of 10 °C/min from room temperature to 1300 °C. The phase composition of the specimens was detected using a MINIFLEX600 X-ray single crystal diffractometer with a Cu target (Rigaku, Tokyo, Japan) (wavelength 0.15406 nm). The scanning speed was 5°/min in the 2θ range of 20–90°. The filament voltage and current were 40 kV and 15 mA, respectively. The microstructure and grain size were observed using an INSPECT F50 scanning electron microscope (FEI, Hillsboro, OR, USA). XPS measurements of the specimens were performed using a XSAM 800 spectrometer (Kratos, Manchester, UK). The density of the specimens was measured using Archimedes’ principle by an ED-300A digital solid density meter (Shanghai Qunlong, Shanghai, China). Hysteresis loops and magnetic properties were measured by a vibrating sample magnetometer (JDAW-2000D, Jilin University, Changchun, China). The Vickers hardness and fracture toughness of the specimens were measured by an HV-30Z Vickers hardness tester (Shanghai Jiezhun, Shanghai, China) at a load of 30 kgf. Fracture toughness was calculated using Equation (1) [71]:

*K_IC_* = 0.0028 (*H_V_*·*P*/*L*)^1/2^ (1)

where *K_IC_* is the fracture toughness (MPa·m^1/2^), *H_V_* stands for indentation hardness (kgf/mm^2^ or N/mm^2^), *L* represents the total crack length (mm), and *P* denotes the applied load (kgf or N). To ensure the accuracy of the Vickers hardness and fracture toughness measurements, six tests were performed on each specimen and averaged.

## 4. Conclusions

In this experiment, SPS was used as the sintering method and metal elements were used as additives to innovatively prepare ultrafine cemented carbide, and the effects of metal element content and sintering parameters on the properties of cemented carbide were investigated, and the results are as follows:

(1) Using SPS combined with metal elements can prepare ultrafine cemented carbide alloys with fine grain size (about 300 nm) and uniform and dense microstructure;
(2) Ultrafine cemented carbide with excellent mechanical properties was prepared at the metal addition of 1.2 wt.% and sintering parameters of 1350 °C, 6 min, and 25 MPa. The Vickers hardness, density, and fracture toughness were *H_V_* 2322.9, 14.25 g/cm^3^, and *K_IC_* 8.7 MPa·m^1/2^, respectively;
(3) Under the optimum conditions (1.2 wt.%, 1350 °C, 6 min, and 25 MPa), the specimens have high magnetic properties, where *M_s_* = 0.487 emu/g, *M_r_* = 0.219 emu/g, and *H_c_* = 283.5 Oe. This indicates that appropriate metal element additions and sintering parameters can effectively reduce the grain size and optimize the microstructure of the alloy;
(4) Compared with the direct addition of grain inhibitors, the addition of the metal elements V and Cr is also able to obtain ultrafine cemented carbides with homogeneous microstructures and excellent mechanical properties, which shows the great potential of using metal elements as additives for the preparation of high-performance cemented carbides. This study provides a new way for the preparation of high performance ultrafine cemented carbide.

The use of spark plasma sintering as the sintering method and metal elements as additives for the preparation of ultrafine cemented carbides in this experiment has shown great potential, and new metal additives and alloy formulations can be explored in the future to further optimize the mechanical properties and microstructure of ultrafine cemented carbides. This includes the improvement of hardness, toughness, wear resistance, and corrosion resistance to meet a wider range of applications.

## Figures and Tables

**Table 1:** Tm and Tp values for each reaction.

Reaction Equation	Tm (°C)	Tp (°C)
4V(s) + 5O_2_(g) = 2V_2_O_5_(s)/4Cr(s) + 3O_2_(g) = 2Cr_2_O_3_(s)	117.4	238.6
Cr_2_O_3_(s) + C(s) = 2CrO(s) + Co(g)↑	731.5	740
V_2_O_3_→V_8_C_7_, Cr_2_O_3_→Cr_3_C_2_	880.0	928.0
V(s) + C(s) = VC(s), 3Cr(s) + 2C(S) = Cr_3_C_2_(s)	1101.7	1141.1

**Table 2:** Composition and elemental content of components in the experiment.

WC (wt.%)	V (wt.%)	Cr (wt.%)	Co (wt.%)
94.0	0.0	0.0	6
93.2	0.4	0.4	6
92.4	0.8	0.8	6
91.6	1.2	1.2	6
90.8	1.6	1.6	6
90.0	2.0	2.0	6

**Table 3:** Melting point of each raw material.

Raw Materials	WC	V	Cr	Co
Melting point (°C)	2870	1890	1907	1495

## Data Availability

Data will be made available on request.
